# Adult‐Onset Primary Pleura Ewing Sarcoma With Recurrent Pleural Effusion: A Case Report and Literature Review

**DOI:** 10.1002/rcr2.70498

**Published:** 2026-02-04

**Authors:** Hamza Khan Khattak, Laiba Malik, Zmarak Ahmed Khan, Mahliqa Kirmani, Saadia Ashraf, Zaryab Bacha, Kamil Ahmad Kamil

**Affiliations:** ^1^ Department of Medicine Khyber Medical College Peshawar Khyber Pakhtunkhwa Pakistan; ^2^ Department of Pulmonology Khyber Teaching Hospital Peshawar Khyber Pakhtunkhwa Pakistan; ^3^ Internal Medicine Department Mirwais Regional Hospital Kandahar Afghanistan

**Keywords:** Askin tumour, Ewing sarcoma, Extraskeletal Ewing sarcoma, pleura, pleural effusion

## Abstract

Extraskeletal Ewing sarcoma (EES) is a rare malignant tumour within the “Ewing family of tumours,” first described by Tefft et al. in 1969. It accounts for less than 1 case per million, typically affecting adolescents and young adults. We report a rare case of pleural EES in a 42‐year‐old woman presenting with chest pain and low‐grade fever persisting for one month. Physical examination showed decreased breath sounds in the right lung base on auscultation. Ultrasound demonstrated right‐sided pleural effusion, an unusual manifestation of EES that can hinder accurate diagnosis. CT scans identified multiple pulmonary nodules, further confirmed by thoracoscopy. Biopsy established the diagnosis of EES. The patient was subsequently treated with multi‐agent VAC/IE chemotherapy. Despite initiation of systemic therapy, the disease followed an aggressive course, and the patient succumbed during follow‐up. We also review existing literature to highlight clinical, pathological, and radiological features of pleural EES, aiming to expand knowledge of this rare presentation.

## Graphical abstract

We present a case of a 42‐year‐old woman presenting with pleural effusion, a rare manifestation of Ewing sarcoma.

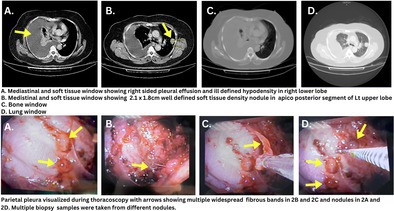

## 1 Introduction

Ewing sarcoma (ES), a malignant tumour primarily affecting bone and soft tissues, such as cartilage and nerves, is the second most common primary bone tumour in children and adolescents [1]. However, a rare form known as Extraskeletal Ewing sarcoma (EES) occurs outside the skeleton and was first described by Tefft et al. in 1969 [2]. It arises from neuroectoderm and has an annual incidence of 0.4 per million [3]. The diverse clinical and histological features of EES often mimic other soft tissue tumours, such as lymphoma and neuroblastoma, creating diagnostic challenges [4]. Additionally, clinical presentations can vary widely based on age, stage, and tumour size [5]. Along with this variability, in tuberculosis‐endemic regions, exudative pleural effusion may be misdiagnosed as tuberculosis (TB), parapneumonic effusion, or lymphoproliferative disease, and recurrent or non‐resolving effusions with pleural masses should prompt biopsy to exclude malignancy [6]. However, instances of non‐diagnostic biopsy can lead to further diagnostic delays. Here, we present a similar case of a 42‐year‐old woman presenting with pleural effusion, a rare manifestation of ES.

## 2 Case Report

A 42‐year‐old female housewife presented to the outpatient department and was admitted with a one‐month history of mild chest pain, low‐grade fever, weight loss, exertional dyspnea, and loss of appetite. She had no known comorbidities or history of smoking. Her temperature, heart rate, and blood pressure were within normal range. Physical examination revealed only decreased breath sounds in the right hemithorax. An abdomino‐pelvic and chest ultrasound found a moderate right‐sided pleural effusion (approximately 1000 mL) and grade 2 fatty liver. Chest X‐ray confirmed effusion. Blood tests (Table 1) were within normal ranges, other than elevated lactate dehydrogenase at 248 U/L (normal range: 91–180 U/L). Blood gas analysis showed reduced PaO_2_ 63 mmHg (normal range: 75.0–100 mmHg), and a minutely elevated pH of 7.48 (normal range: 7.35–7.45). The transthoracic echocardiogram documented mild tricuspid regurgitation and pulmonary arterial hypertension on Doppler.

**TABLE 1:** Laboratory and Diagnostic Findings.

Parameter	Observed value	Normal range	Parameter	Observed value	Normal range
*Haematology*			*Biochemistry*		
(a) Complete Blood Count (CBC)			(a) Renal Function Tests (RFTs)		
WBC	9.48 × 10^3^/μL	4–11 × 10^3^/μL	Blood urea	24.9 mg/dL	10–50 mg/dL
RBC	4.85 × 10^6^/μL	4–6 × 10^6^/μL	Creatinine	0.75 mg/dL	0.42–1.06 mg/dL
Haemoglobin	13.3 g/dL	11.5–17.5 g/dL	(b) Liver Function Tests (LFTs)		
Haematocrit	39.9%	36%–54%	LDH	248 U/L	91–180 U/L
MCV	82.3 fL	76–96 fL	Total Bilirubin	0.21 mg/dL	0.1–1.0 mg/dL
MCH	27.4 pg	27–33 pg	ALT/GPT	27.8 U/L	10–50 U/L
MCHC	33.3 g/dL	33–35 g/dL	Alkaline Phosphatase	88 U/L	35–104 U/L
Platelets	402 × 10^3^/μL	150–450 × 10^3^/μL	(c) Inflammatory Marker		
MPV	10.4 fL	7.2–11 fL	CRP	7.76 mg/L	< 5.0 mg/L
Neutrophils	69.6%	40%–75%	(d) Electrolytes		
Lymphocyte	23.5%	20%–45%	Sodium	134 mmol/L	135–150 mmol/L
Monocytes	4.4%	2%–10%	Potassium	4.5 mmol/L	3.5–5.1 mmol/L
(b) Coagulation Profile			Chloride	108 mmol/L	96–112 mmol/L
PT	13 s	12 s	*Echocardiogram*		
aPTT	34 s	30 s	Fractional Shortening	33%	> 27%
INR	1.09	—	Ejection Fraction	63%	50%–70%
D‐DIMER	430 ng/mL	< 500 ng/mL	RVP (Sys)	35 mmHg	—
*Blood gas analysis*					
pH	7.475 ↑	7.350–7.450	HCO_3_^−^	27.6 mmol/L	22–28 mmol/L
pCO_2_	38.4 mmHg	35–45 mmHg	O_2_ Saturation	93.5%	> 95%
pO_2_	62.9 mmHg ↓	75–100 mmHg			

**FIGURE 1:**
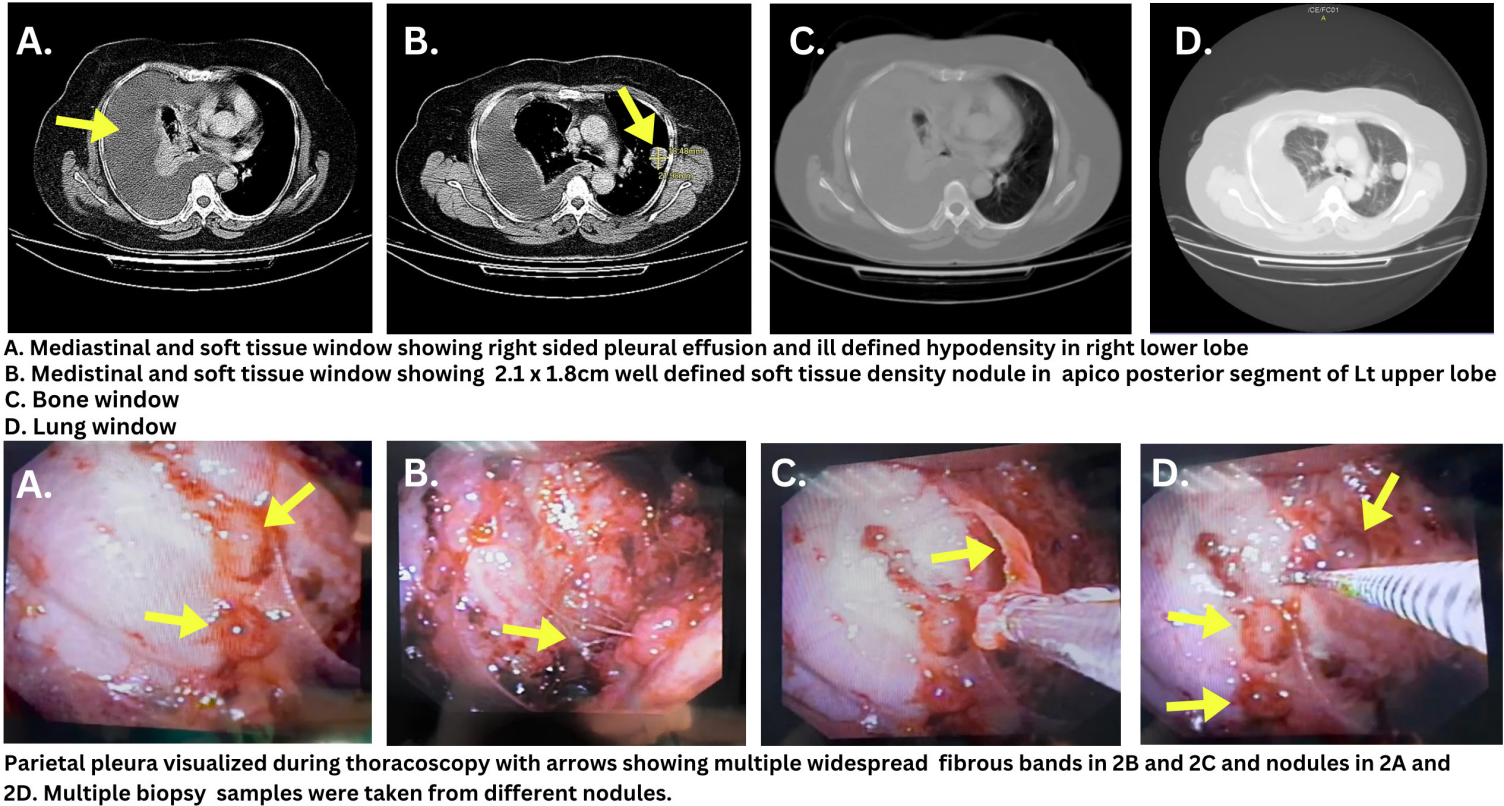
Axial contrast‐enhanced chest CT with corresponding thoracoscopic views showing moderate right‐sided pleural effusion with collapse consolidation in the right lower lobe, along with a solitary soft tissue nodule in the left upper lobe. Thoracoscopy revealed multiple widespread pleural nodules, from which biopsies and brushings were obtained.

Pleural fluid was drained via thoracentesis, and cytology showed a lymphocytic exudative picture, raising suspicions for tuberculosis. Chest Computed Tomography (CT) with contrast imaging revealed a poorly defined, hypodense mass located in the collapsed, consolidated right lower lobe. Additionally, a well‐defined solitary soft tissue density nodule measuring 2 × 1.5 cm was identified in the apical‐posterior segment of the left upper lobe (Figure 1). To evaluate these lesions, a closed (needle) pleural biopsy under local anaesthesia was performed, and the patient was discharged due to improvement in her condition, with instructions to follow up with biopsy results. Histopathology reports revealed nondiagnostic fibroskeletal and fibroadipose tissue with fibrosis and acute on chronic inflammation. Acid‐fast bacilli for TB or other fungi/microorganisms came out negative. Additionally, the sample did not contain any synaptophysin, a protein that indicates cancer cells are absent.

The patient was readmitted approximately 2 weeks later for reevaluation due to non‐specific findings. A video‐assisted thoracoscopic pleural biopsy was performed via a flexible pleuroscope (single‐port thoracoscopy) under local anaesthesia from the 6th intercostal space on the anterior axillary line. During the procedure, another 1 L of pleural fluid was aspirated through the suction tube. Direct visualisation during thoracoscopy identified numerous nodules and widespread fibrous bands previously not appreciated on CT imaging. Biopsy samples were taken from different nodules and sent for histopathology. Additionally, pleurodesis with talc powder was performed to prevent any recurring pleural effusion. The patient's C‐reactive protein remained uptrending despite escalation of antibiotics; however, the patient was discharged.

**FIGURE 2:**
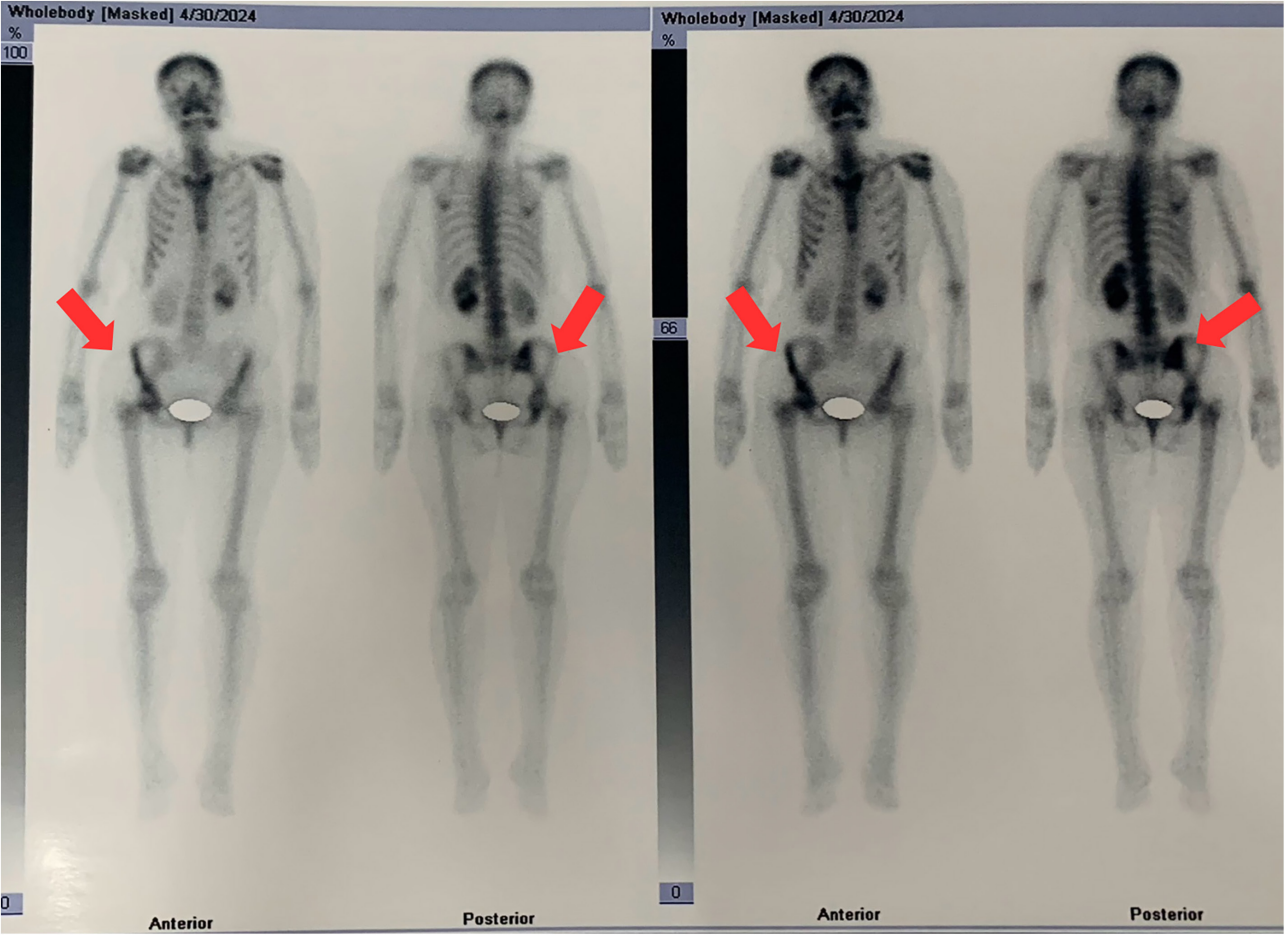
Whole‐body bone scan with Tc‐99 m MDP showing no metastatic bone disease. Mild non‐homogeneous uptake in the right hip joint likely reflects arthritic changes; the rest of the skeleton shows uniform, symmetrical tracer uptake.

The histopathological report of the biopsy samples this time revealed a pattern of atypical small round blue cells with a lobular architecture, characteristic of ES. The following histochemical stains were assessed: TTF1 (Negative), INSM1 (Negative), Desmin (Negative), CK (Weak), NKX2.2 (Patchy positive). Later, a whole‐body bone scan with the help of Technetium‐99m methylene diphosphonate (Tc‐99m MDP) showed no scintigraphic evidence of metastatic bone disease except for some non‐homogeneous uptake of the tracer in the right hip joint, which was most likely due to arthritic changes involving the right hip joint (Figure 2). The rest of the skeleton showed uniform and bilaterally symmetrical tracer uptake. Based on the above results, the patient was finally diagnosed with EES in the pleura.

The patient was transitioned from the pulmonology ward to the oncology hospital, where she was receiving VAC/IE (i.e., vincristine, doxorubicin, cyclophosphamide, etoposide, ifosfamide, and prednisone) chemotherapy regimen as treatment protocol for ES. Despite the initiation of multi‐agent chemotherapy (VAC/IE protocol) once the diagnosis was established, the disease course remained aggressive. The patient ultimately succumbed at the seventh month of follow‐up, reflecting poor prognosis. This case emphasises the diagnostic difficulties, the consequences of delayed recognition, and the limited effectiveness of current therapeutic options in achieving long‐term survival in such rare and aggressive tumours.

## 3 Discussion

Ewing sarcoma, first described by James Ewing in 1921 as a type of primary bone tumour, is now recognised as a broader group of tumours with similar morphology, including classic Ewing osteosarcoma, EES, Askin tumour (arising in the chest and lung region), and soft tissue‐based primitive neuroectodermal tumours (PNET) [7]. ES commonly occurs in individuals with a mean age of 15 years and accounts for less than 5% of all soft tissue sarcomas [1]. Common extra‐skeletal sites include the chest wall, paraspinal region, retroperitoneum, kidney, ovary, and cervix [8].

In our case, EES occurred in the pleura and presented with recurrent pleural effusion, an unusual finding that can easily be misdiagnosed. This is especially true in tuberculosis‐endemic regions, where exudative effusions are often assumed to be tuberculous [9]. Literature reports diagnostic challenges in such cases, with initial chest X‐ray findings showing only effusion and pleural cytology being negative for malignant cells [10]. Pleural fluid cytology is shown to have limited sensitivity for malignant pleural effusion (approximately 58% overall) and can be particularly insensitive for small round cell tumours [11]. Using a cell‐block preparation with immunohistochemistry (IHC) improves diagnostic yield, but a tissue biopsy is still required for a definitive diagnosis [12], as seen in our case. Therefore, suspicion should be raised in patients with recurrent or atypical effusions, non‐resolving collections despite therapy, or imaging evidence of a pleural‐based mass. In such cases, image‐guided or thoracoscopic biopsy requiring histopathology is critical to exclude mimics such as adenocarcinoma, lymphoma, rhabdomyosarcoma, or neuroblastoma.

**FIGURE 3:**
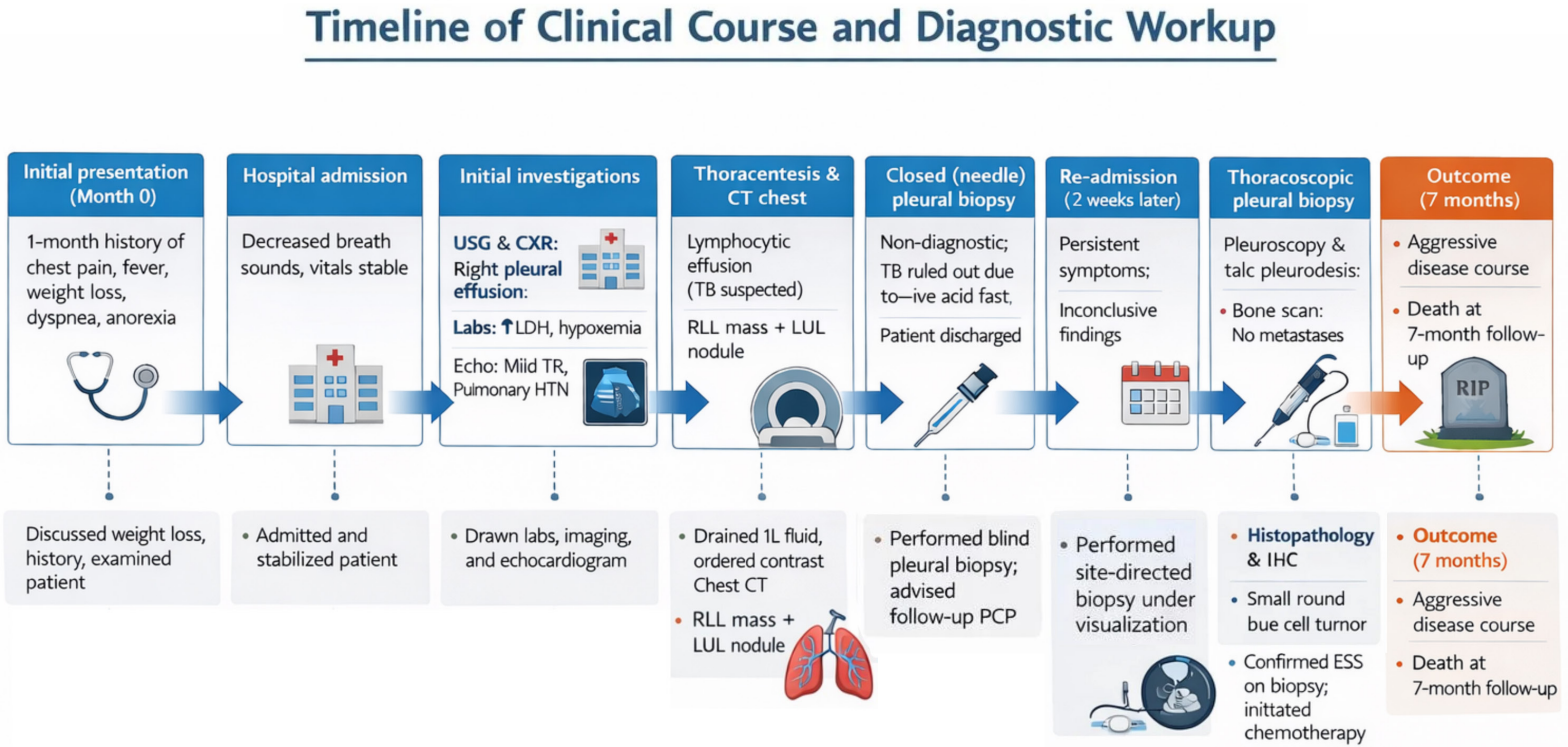
Timeline summarising the clinical and diagnostic course of the patient (illustration created with BioRender.com).

Using the keywords “EES,” “Ewing Sarcoma of the Pleura,” and “Pleural Effusion,” relevant articles were retrieved from MEDLINE (PubMed), EMBASE, Web of Science, and Google Scholar. Between 2002 (first reported case) and 2025, nine cases of EES with pleural effusion were reported [13, 14, 15, 16, 17, 18, 19, 20, 21] (Table 2), including 5 males and 4 females (mean age 20.5 years). Compared with prior reports, which predominantly involve children and young adults, our 42‐year‐old patient may represent the oldest reported case to date, to our knowledge. The clinical and diagnostic course of our patient is summarised in Figure 3 for comparison with previously reported cases. Her subacute chest discomfort, fever, dyspnea, and large exudative effusion resembled most prior cases [13, 14, 15, 17, 18, 19], differing from the slow‐growing, effusion‐negative mass reported by Tsunezuka et al. [16]. Imaging in previous cases often showed rib and chest wall involvement [13, 14, 18], whereas our patient had no bony erosion but was uniquely presented with a contralateral solitary pulmonary nodule. Similar to Zou et al. [18], thoracoscopy revealed multifocal pleural nodules, emphasising its value when CT underestimates surface disease. Pleural fluid cytology was non‐diagnostic [14], requiring tissue biopsy. Staging showed no osseous metastasis, unlike the nodal and vertebral involvement reported by Bhaskaran et al. [19]. Treatments varied in the literature: five patients received chemotherapy, one underwent thoracoscopic resection with no recurrence at one year, and two did not specify treatment.

**TABLE 2:** Literature review of similar case reports.

Study	Patient (Age/sex)	Location and imaging (mass + effusion)	Clinical manifestations	Diagnosis	Therapy	Outcome
Wolf et al. [13]	17/Male	6th rib soft tissue mass (6 cm), massive effusion	Dyspnea, Chest pain	Needle Biopsy	NS	NS
Ozge et al. [14]	18/Female	R hemithorax 7 × 4 cm mass, diffuse effusion, rib erosions	Back pain, cough and dyspnea	Needle biopsy	NS	NS
Kushwaha [15]	20/Female	19 × 12 × 9 cm L pleural/rib mass, hemorrhagic effusion	Fever, Lt sided chest pain, dyspnea	Transthoracoscopy and pleural needle biopsy	Chemotherapy	NS
Tsunezuka et al. [16]	27/Female	Pleural mass grew 2 → 9 cm, no effusion	No obvious symptoms	Thoracoscopic resection and pathological exam	Thoracoscopic resection	No recurrence after 1 year
Mathew et al. [17]	7/Male	Multiple pleural masses, large L effusion, mediastinal shift	Dyspnea	Needle biopsy	Chemotherapy	NS
Zou et al. [18]	14/Male	Huge R chest mass, bloody effusion, pleural nodules	Dyspnea, fever, disappeared breath sounds	Thoracoscopic biopsy	Radiotherapy, Chemotherapy	Death
Bhaskaran et al. [19]	34/Female	13 × 11 × 15 cm R middle lobe mass, mild effusion	Fever, cough with phlegm, right chest, and back pain	Needle biopsy	Surgery, Radiotherapy, Chemotherapy	NS
Wu et al. [20]	11/Male	Pleural mass (NS)	Left shoulder pain	Needle biopsy	Chemotherapy	Pain relief
Alkoheji et al. [21]	31/Male	Multifocal pleural masses (13 × 11 × 15 cm R), invasive pulmonary artery thrombosis, mild R pleural effusion	Productive cough with white sputum and blood streaks	CT‐guided Tru‐Cut biopsy	Palliative care only	NS

Abbreviation: NS, not stated.

Definitive EES diagnosis relies on imaging, histopathology, and IHC of tumour samples. Microscopically, small, round tumour cells exhibit sparse cytoplasm and round‐to‐oval nuclei, arranged in lobules separated by vascular fibrous tissue. Electron microscopy reveals minimal organelles and abundant cytoplasmic glycogen granules [18].

Histopathologically, markers such as CD99 and FLI‐1 are widely accepted for the diagnosis of ES; however, they are seen to have variable accuracy. NKX2.2, a homeodomain transcription factor, demonstrates promising sensitivity (93%) and specificity (89%) for EES diagnosis [22]. It is diffusely positive in EES tissues, significantly more so than in other small round cell tumours, making it a potential novel marker for differential diagnosis of EES [23]. Immunohistochemical staining for S‐100 protein, neurofilament protein, GFAP, desmin, F8, UEA‐1, and keratin is typically negative [14] which aids in ES diagnosis by excluding other (myogenic, neural crest, neuroendocrine) lineages or differentiation pathways. Together, the combination of CD99 and NKX2.2 provides a strong diagnostic basis [23]. Furthermore, molecular testing with FISH or RT‐PCR can also be used to detect EWSR1 gene rearrangement, which is the hallmark of ES [24]. This is typically the t(11;22) translocation, which creates the EWS‐FLI1 fusion oncoprotein that drives the tumour pathogenesis [24].

Imaging on a CT scan typically reveals heterogeneous enhancement with hypoattenuating areas indicating necrosis, along with high‐density foci suggesting haemorrhage in affected regions [21]. Although the findings are not specific, chest CT is essential for assessing tumour size, extent, and boundaries of the EES tumour and plays a key role in guiding management.

The treatment for EES mirrors that of ES and includes aggressive surgical resection, radiation therapy, and multi‐agent chemotherapy. Standard regimens involve alternating cycles of vincristine, doxorubicin, cyclophosphamide (VDC), and ifosfamide with etoposide (IE). Prognostic factors include tumour size, complete surgical resection with wide margins, initial chemotherapy response, presence of metastases, extent of necrosis, and EWS‐FLI1 transcript levels [25].

Hence, a definitive diagnosis of pleural EES requires close collaboration among radiologists, pathologists, thoracic specialists, and oncologists to avoid misdiagnosis and to distinguish it from more common pathologies that may delay timely recognition. Clinicians should maintain suspicion for rare malignancies in young adults presenting with recurrent “idiopathic” pleural effusions, particularly when initial less‐invasive investigations are non‐diagnostic. Thoracoscopy is recommended for direct visualisation and adequate tissue sampling to guide therapeutic decisions. Early detection and prompt initiation of systemic chemotherapy remain essential for improving survival in this aggressive malignancy. Continued reporting of cases and larger series is needed to expand the evidence base, refine diagnostic strategies, and optimise management of atypical pleural EES presentations, ultimately leading to better patient outcomes.

## Author Contributions

Hamza Khan Khattak and Laiba Malik contributed to data collection, patient history documentation, and drafting of the initial manuscript. Mahliqa Kirmani and Zmarak Ahmed Khan assisted in the literature review and interpretation of diagnostic findings. Saadia Ashraf supervised the clinical management of the patient, provided critical revisions, and validated the diagnostic approach. Zaryab Bacha contributed to manuscript editing, formatting, and final approval of the draft. Kamil Ahmad Kamil assisted in reviewing the literature, refining the discussion, submission, and critically revising the manuscript for intellectual content. All authors reviewed and approved the final version of the manuscript and agree to be accountable for its content.

## Ethics Statement

This case report was conducted in accordance with the principles of the Declaration of Helsinki. Formal ethical approval was not required as this is a single case report without experimental intervention. Written informed consent for publication of clinical details and images was obtained from the patient's legal guardian/family. All efforts have been made to ensure patient anonymity, and no identifiable information has been disclosed.

## Consent

The authors declare that written informed consent was obtained for the publication of this manuscript and accompanying images and attest that the form used to obtain consent from the patient complies with the Journal requirements as outlined in the author guidelines.

## Conflicts of Interest

The authors declare no conflicts of interest.

## Data Availability

Data sharing not applicable to this article as no datasets were generated or analysed during the current study.
